# High testosterone levels in prostate tissue obtained by needle biopsy correlate with poor-prognosis factors in prostate cancer patients

**DOI:** 10.1186/1471-2407-14-717

**Published:** 2014-09-26

**Authors:** Yasuhide Miyoshi, Hiroji Uemura, Susumu Umemoto, Kentaro Sakamaki, Satoshi Morita, Kazuhiro Suzuki, Yasuhiro Shibata, Naoya Masumori, Tomohiko Ichikawa, Atsushi Mizokami, Yoshiki Sugimura, Norio Nonomura, Hideki Sakai, Seijiro Honma, Masaoki Harada, Yoshinobu Kubota

**Affiliations:** Department of Urology, Yokohama City University Graduate School of Medicine, Yokohama, Japan; Department of Biostatistics and Epidemiology, Graduate School of Medicine, Yokohama City University, Yokohama, Japan; Department of Urology, Gunma University Graduate School of Medicine, Maebashi, Japan; Department of Urologic Surgery and Andrology, Sapporo Medical University School of Medicine, Sapporo, Japan; Department of Urology, Chiba University Graduate School of Medicine, Chiba, Japan; Department of Integrative Cancer Therapy and Urology, Kanazawa University Graduate School of Medical Science, Kanazawa, Japan; Department of Nephro-Urologic Surgery and Andrology, Mie University Graduate School of Medicine, Tsu, Japan; Department of Urology, Osaka University Graduate School of Medicine, Osaka, Japan; Department of Nephro-urology, Nagasaki University Graduate School of Biomedical Sciences, Nagasaki, Japan; ASKA Pharma Medical Co., Ltd, Kawasaki, Japan; Department of Urology and Pathology, Kanagawa Cancer Center, Asahi-ku, Yokohama, Japan

**Keywords:** Prostate cancer, Androgen, Testosterone, Dihydrotestosterone

## Abstract

**Background:**

There is currently no consensus on the correlations between androgen concentrations in prostate tissue and blood and stage and pathological grade of prostate cancer. In this study, we used a newly-developed ultra-sensitive liquid-chromatography tandem mass spectrometry method to measure testosterone (T) and dihydrotestosterone (DHT) concentrations in blood and needle biopsy prostate specimens from patients with prostate cancer.

**Methods:**

We analyzed androgen levels in 196 men diagnosed with prostate cancer. All patients had undergone systematic needle biopsy, and an additional needle biopsy from the peripheral zone was conducted for the simultaneous determination of T and DHT. We analyzed the relationships between T and DHT levels in tissue and blood and Gleason score, clinical stage, and percentage of positive biopsy cores, using multivariate analysis.

**Results:**

The median T and DHT levels in blood were 3551.0 pg/mL and 330.5 pg/mL, respectively. There was a strong correlation between serum T and DHT. The median T and DHT levels in prostate tissue were 0.5667 pg/mg and 7.0625 pg/mg, respectively. In multivariate analysis, serum prostate-specific antigen and tissue T levels were significantly associated with poor prognosis; high T levels in prostate tissue were significantly related to high Gleason score (p = 0.041), advanced clinical stage (p = 0.002), and a high percentage of positive biopsy cores (p = 0.001).

**Conclusions:**

The results of this study indicate that high T levels in prostate tissue are related to high Gleason score, advanced clinical stage, and a high percentage of positive biopsy cores in patients with prostate cancer. T level in needle biopsy specimens may therefore be a useful prognostic factor in prostate cancer patients.

**Electronic supplementary material:**

The online version of this article (doi:10.1186/1471-2407-14-717) contains supplementary material, which is available to authorized users.

## Background

Prostate cancer is the most common internal cancer and the second most frequent cause of cancer-related deaths among men in the United States. Although the incidence of prostate cancer in Japan is lower than in the United States, it has been gradually increasing in recent years. The etiology of prostate cancer is unclear, but it is thought to be multifactorial, with genetic, dietary, and environmental causes. Although prostate cancer initially responds to androgen ablation therapy, most patients ultimately become hormone-refractory and show treatment failure.

The ability to predict prostate tumor behavior is important, because more intensive treatment is necessary to prevent the development of castration-resistant prostate cancer (CRPC). Pathological grade and clinical stage can strongly predict tumor aggressiveness, but no useful molecular markers have yet been identified. Several previous studies have reported blood and prostate tissue levels of testosterone (T) and dihydrotestosterone (DHT) in patients with prostate cancer, but these studies have involved small sample sizes, and several have measured the levels using radioimmunoassays (RIAs), which require a large amount of tissue (≥20 mg) for androgen measurement [1, 2]. There have been few reports regarding the correlation between prostate cancer aggressiveness and androgen concentrations measured in smaller prostate tissue samples, such as those obtained by needle biopsy.

Advancements in liquid-chromatography tandem mass spectrometry (LC-MS/MS) methods mean that T and DHT levels can be measured in small tissue samples with high sensitivity and reliability [3–6]. LC-MS/MS can be used to measure androgen concentrations in tissue samples as small as those obtained by a single needle biopsy (approximately 3 mg), and the latest LC-MS/MS technique is more than 10 times as sensitive as the RIAs used in the past, especially in the lower concentration range [7].

Previous reports revealed that T levels were higher and DHT levels lower in prostate cancer tissues compared with tissues from patients with benign prostatic hyperplasia, although there is currently no consensus on androgen concentrations in prostate cancer tissues from men with different stages and with different pathological grades of disease [6, 8–10]. Moreover, the relationship between tissue androgen concentrations and tumor behavior in prostate cancer is not clear. In the present study, we measured androgen (T and DHT) levels in blood and prostate tissues using LC-MS/MS and analyzed the correlations between these levels and prognostic factors in patients with prostate cancer.

## Methods

### Patients

A total of 359 patients with suspected prostate cancer underwent prostate needle biopsy for primary pathological diagnosis at major cancer treatment facilities in Japan between April 2000 and July 2003. Blood samples were also collected. All blood samples were taken between 09.00 h and 15.00 h to minimize the effect of daily T variations. Patients underwent a systematic needle biopsy. An additional needle biopsy sample was taken from the peripheral zone of the prostate as a chemical biopsy, for the simultaneous determination of T and DHT. The tissues were immediately frozen at -70°C. Of the 359 patients, 163 were shown not to have cancer and data for the remaining 196 men diagnosed with prostate cancer were analyzed. The patient characteristics are shown in Table 1.

**Table 1:** **Patient characteristics**

No. patients	196
Age, years (mean, SD)	70.6 (7.334)
PSA, ng/mL (median, 95% CI)	11.5 (32.43–82.73)
Prostate volume, cm^3^ (median, 95% CI)	27.7 (29.83–34.91)
Gleason score ≤7, ≥8 (%)	130 (67.4), 63 (32.6)
Clinical stage ≤ III, ≥IV (%)	166 (86.0), 27 (14.0)
% Positive core <30%, ≥30% (%)	143 (74.1), 50 (25.9)

*SD*: Standard deviation; *PSA*: Prostate-specific antigen; *CI*: Confidence interval.

T and DHT concentrations in prostate tissues and blood were determined by LC-MS/MS. The method was validated to ensure that the result was within the 20% range for accuracy and precision [7]. The determination limit of the method was 0.5 pg/shot for T and 1 pg/shot for DHT. The concentrations of T and DHT were subsequently expressed in pg/mg.

We analyzed the relationships between T and DHT levels in prostate tissue and blood and prognostic factors including Gleason score, clinical stage, and percentage of positive biopsy cores (% positive cores) using multivariate analysis. All prostate biopsy samples were reviewed by a central pathologist. Informed consent was obtained from all patients and the experimental procedures were conducted in accord with the ethical standards of the Helsinki Declaration. This study was approved by each of the participating institution’s review boards (Additional file 1).

### Biological samples

Each needle chemical biopsy prostate sample (2–8 mg) from patients with prostate cancer was placed in a microtube and frozen immediately in liquid nitrogen or in a dry-ice box, and then stored at -70°C until hormone analysis. Serum samples were separated from blood and stored at -70°C until analysis.

### Chemicals and materials

T, DHT, [16,16,17α-^2^H_3_]-T (T-d_3_), and [16,16,17α- ^2^H_3_]-DHT (DHT-d_3_) were purchased from Sigma-Aldrich (St. Louis, MO, USA). The Bond Elut C_18_ cartridge was purchased from Varian (Palo Alto, CA, USA), and 4-dimethylaminopyridine (DAP), 2-methyl-6-nitrobenzoic anhydride (MNBAn), and picolinic acid (PA) were purchased from Tokyo Kasei Industry (Tokyo, Japan). Triethylamine (TEA) was purchased from Wako Pure Chemical Industries (Osaka, Japan). The Cadenza CD C-18 columns and Capcell Pak SCX UG80 pre-columns were purchased from Intact (Kyoto, Japan) and Shiseido (Tokyo, Japan), respectively.

The derivatization reagent was prepared as follows: 10 mg DAP, 20 mg MNBAn, and 25 mg of PA were dissolved in 1 mL of tetrahydrofuran and the mixture was agitated until it became cloudy or crystals appeared. The reagent solution was used after 3–5 min [5]. Serum prostate-specific antigen (PSA) levels were measured using a DPC Imrise third generation PSA assay kit.

### LC-MS/MS

Serum hormone levels were determined by LC-MS/MS, as described by Yamashita et al. [5]. T and DHT levels in prostate tissue samples were measured using an API-5000 triple-stage quadrupole mass spectrometer (Applied Biosystems, Foster City, CA, USA) connected to a Shimadzu LC-20 AD pump and Shimadzu SIL HTC autosampler (Shimadzu, Kyoto, Japan) and an electrospray ionization (ESI) ion-source device. The columns used were a Capcell Pak SCX UG80 pre-column (35 mm × 2 mm internal diameter, particle size 5 μm) and a Cadenza CD-C_18_ analytical column (150 mm × 3 mm internal diameter, particle size 3 μm), which had been maintained at 40°C.

The mobile phase consisted of CH_3_CN-MeOH (50:50, v/v) (solvent A) and 0.1% formic acid (solvent B). For gradient elution, A/B was used at a ratio of 60/40–90/10 between 0 and 5.0 min, and 90/10–100/0 between 5.0 and 7.0 min. Solvent A alone was used between 7.0 and 9.0 min. A/B was used at 100/0–60/40 between 9.0 and 11 min. The flow rate was 0.4 mL/min. The following ESI conditions were used: spray voltage, 3,300 V; collision gas, nitrogen, 45 psi; curtain gas, 11 psi; ion source temperature, 600°C; and ion polarity, positive.

### Tissue hormone analysis

Each frozen prostate tissue sample (1–3 mg, 5–13 mm) was weighed and then a piece of the sample was cut off with scissors. Purified water (0.5 mL) was added to the cut sample, which was then homogenized for 20 s using an Ultra-Turrax homogenizer with an ice-cooling bath, and then washed with 0.5 mL water. Ethanol (3 mL) and 100 pg of T-d_3_ and DHT-d_3_ were added to the homogenate and the mixture was shaken at 50°C for 2 h. The solution was allowed to stand at 4°C overnight to allow complete precipitation of the protein. After centrifugation (4°C, 3,000 rpm, 10 min), the supernatant was isolated and the solvent was evaporated using a centrifugation evaporator. The residue was dissolved in methanol (0.25 mL), diluted with purified water (1 mL), and loaded onto a Bond Elut C_18_ cartridge pre-conditioned with methanol (6 mL) and purified water (6 mL). The cartridge was washed with purified water (1 mL) followed by 30% acetonitrile solution (v/v, 3 mL). T and DHT were subsequently eluted with 70% acetonitrile solution (v/v, 3 mL) and the solvent was removed using a centrifugation evaporator.

The residue was dissolved in 100 μL reagent mixture, prepared as described above. TEA (20 μL) was added and the resulting mixture was allowed to stand at room temperature for 30 min. After dilution of the reaction mixture with 1% acetic acid solution (v/v, 1 mL) to stop the reaction, the resulting derivative was loaded onto a Bond Elut C_18_ cartridge pre-conditioned with methanol and purified water. The cartridge was washed with purified water (1 mL) followed by 40% acetonitrile solution (v/v, 3 mL). The derivatives were then eluted with 80% acetonitrile solution (v/v, 3 mL). The solvent was evaporated to dryness using a centrifugation evaporator at 53–55°C, the residue was dissolved in 40% acetonitrile solution (v/v, 100 μL), and a 40-μL aliquot of the solution was subjected to LC-ESI-MS/MS.

### Validation of analytical methods

The inter- and intra-assay accuracies and precisions of the LC-MS/MS were tested as described by Shibata et al. [11].

### Cancerous and noncancerous regions in the prostate

Hormone-naïve prostate cancer specimens were obtained from 10 patients by radical prostatectomy. Each prostate specimen was cut into two mirror-image 2.5-mm-thick fragments, each of which was cut into a further 90 sections. All 90 sections were used for the androgen assays and histopathology diagnosis, to analyze the relationships between tissue androgen concentrations with histopathological findings. There was no significant difference in tissue T or DHT levels between cancerous and noncancerous sections of individual prostate cancer sections (mean T levels, p = 0.796; mean DHT levels, p = 0.912). The tissues used for androgen concentration measurements in this study were not examined by a pathologist, because the whole of the samples were used for androgen measurement. Cancer diagnosis was based on other, simultaneously-obtained biopsy specimens. These results indicated that the results of androgen measurements were unaffected by the use of cancerous or noncancerous lesions.

### Histopathology

All prostate biopsy samples were reviewed by a central pathologist. Pathological grading was performed according to the Gleason classification system. Histological diagnoses were made by the central pathologist blinded to the patients’ serum and prostate androgen measurements.

### Statistical analysis

We compared tissue and blood androgen levels using Pearson’s correlation coefficient. If some factors were found to be correlated with other factors, the correlated factors were not analyzed simultaneously because of multicollinearity. We also analyzed the relationships between T and DHT levels in tissue and blood and prognostic factors including Gleason score, clinical stage, and % positive cores by multivariate analyses using a logistic regression model. All data were analyzed using IBM SPSS ver. 21 software. Each test was two-sided, and p values <0.05 were considered significant.

## Results

### Androgen levels in blood and prostate tissue obtained by needle biopsy

The androgen levels in prostate tissue and peripheral blood are shown in Table 2. serum T levels were almost 10-fold higher than DHT levels in peripheral blood, whereas DHT levels in prostate tissue were almost 10-fold higher than tissue T levels. These serum and tissue androgen levels were similar to those in other reports concerning prostate cancer patients.

**Table 2:** **Androgen levels in blood and prostate tissue of patients with prostate cancer (n = 196)**

T (blood), pg/mL (median, 95% CI)	3551.0 (3499.8–3902.2)
DHT (blood), pg/mL (median, 95% CI)	330.5 (333.2–382.3)
T (tissue), pg/mg (median, 95% CI)	0.5667 (0.9401–1.3820)
DHT (tissue), pg/mg (median, 95% CI)	7.0625 (8.7513–11.6266)

*T*: Testosterone; *CI*: Confidence interval; *DHT*: Dihydrotestosterone.

Correlations between T and DHT levels in serum and prostate tissue are shown in Table 3. There were strong correlations between T and DHT blood levels (Pearson correlation coefficient: 0.76996) and we therefore did not analyze these two factors simultaneously, because of multicollinearity.

**Table 3:** Correlations between testosterone and dihydrotestosterone in serum and prostate tissue*

	T (blood)	T (tissue)	DHT (blood)	DHT (tissue)
T (blood)	1	0.00901	0.76996	0.08784
T (tissue)	0.00901	1	0.02471	0.25907
DHT (blood)	0.76996	0.02471	1	0.16242
DHT (tissue)	0.08784	0.25907	0.16242	1

*Pearson correlation coefficients.

### Gleason score

The relationships between Gleason score and androgen levels and other clinical factors are shown in Table 4. Logistic regression identified high serum PSA (hazard ratio [HR]: 1.022, p = 0.001), low blood T levels (HR: 0.706, p = 0.012) and high tissue T levels (HR: 1.388, p = 0.041) as factors significantly associated with high Gleason score.

**Table 4:** Correlation between prognostic factors and androgen concentrations in blood and prostate tissue*

	Gleason score					Clinical stage					% positive cores				
Parameter	≥8	≤7	p value	HR	95% CI	≥IV	≤III	p value	HR	95% CI	≥30%	<30%	p value	HR	95% CI
No. of patients	63	130				27	166				50	143			
Median age (SD), years	73.0 (7.1)	69.5 (7.2)	0.076	1.050	0.995–1.108	75.0 (7.3)	70.0 (7.2)	0.255	1.049	0.966–1.138	73.0 (7.4)	70.0 (7.1)	0.120	1.052	0.987–1.121
Median serum PSA (95% CI), ng/mL	32.1 (69.2-216.2)	8.76 (12.3-20.2)	0.001	1.022	1.009–1.036	55.9 (113.4-435.8)	9.68 (16.5-27.9)	0.010	1.009	1.002–1.017	52.8 (83.6-262.0)	8.61 (9.6-24.8)	0.005	1.017	1.005–1.030
Median prostate volume, mL	27.0 (28.5-40.0)	27.9 (28.8-34.0)	0.387	0.991	0.970–1.012	33.2 (31.7-55.1)	27.5 (28.3-32.8)	0.057	1.024	0.999–1.049	28.4 (29.3-41.0)	27.6 (28.6-34.1)	0.164	0.982	0.957–1.007
Median concentration of T (95% CI) (blood) (/1000), pg/mL	3.11 (2.90-3.62)	3.64 (3.67-4.14)	0.012	0.706	0.538–0.926	3.21 (2.56-4.06)	3.61 (3.56-3.96)	0.354	0.839	0.579–1.216	3.12 (2.84-3.74)	3.66 (3.62-4.06)	0.113	0.78	0.575–1.060
Median concentration of DHT (95% CI) (tissue), pg/mg	5.31 (6.15-10.27)	7.31 (9.26-13.02)	0.222	0.974	0.935–1.016	4.68 (3.94-11.16)	7.28 (9.04-12.18)	0.756	0.992	0.942–1.044	5.26 (4.86-7.32)	7.58 (9.77-13.46)	0.003	0.878	0.806–0.956
Median concentration of T (95% CI) (tissue), pg/mg	0.90 (1.20-2.23)	0.46 (0.68-1.09)	0.041	1.388	1.013–1.900	2.03 (1.74-3.84)	0.50 (0.72-1.06)	0.002	1.713	1.227–2.391	1.03 (1.42-2.70)	0.47 (0.67-1.02)	0.001	2.432	1.425–4.148

*Multivariate analysis by logistic regression. *SD*: Standard deviation; *HR*: Hazard ratio; *CI*: Confidence interval.

### Clinical stage

High serum PSA levels (HR: 1.009, p = 0.010) and high tissue T levels (HR: 1.713, p = 0.002) were significantly related to advanced clinical stage (Table 4).

### % Cancer-positive cores

High serum PSA (HR: 1.017, p = 0.005), low tissue DHT (HR: 0.878, p = 0.003) and high tissue T levels (HR: 2.432, p = 0.001) were significantly related to a high % positive cores (Table 4).

In summary, multivariate analysis demonstrated that high serum PSA and high tissue T levels were significantly associated with poor-prognosis factors such as high Gleason score, advanced clinical stage, and high % positive cores in men with prostate cancer.

## Discussion

The current study demonstrated associations between high serum PSA and high tissue T levels and poor-prognosis factors, including high Gleason score, advanced clinical stage and high % positive cores, in men with prostate cancer. To ensure that histological examination and androgen measurement were conducted simultaneously, we measured androgen levels in 90 tissue samples from 10 prostatectomy specimens. Each specimen was cut into two 2.5-mm-thick mirror-image fragments for androgen assay and histopathological diagnosis, respectively, allowing direct analysis of the relationship between androgen levels and histopathological findings. Further, we found no difference in tissue T levels between cancerous and noncancerous sections of individual prostate cancer specimens. This indicates that not only prostate cancer cells themselves but also their surrounding cells might be responsible for the androgen biosynthesis environment in prostate cancer tissues. In this study, we measured androgen levels in single needle prostate cancer biopsies, but did not confirm if the specimen used for androgen measurement was cancerous or noncancerous. However, the above results suggest that the results of the androgen assay would not be influenced by the presence or absence of cancerous tissue in the sample.

Several studies of androgen concentrations in prostate tissues have used bulk tissues such as prostatectomy specimens, and androgen levels have been measured by RIA methods [3, 4, 9, 12–14]. However, the present study demonstrated that: (1) these measurements could be conducted using the small amounts of tissue obtained by needle biopsy, rather than excised prostate tissues; and (2) androgen measurements could be carried out much more precisely than by RIA, by using LC-MS/MS. Nishiyama and colleagues used LC-MS/MS and reported that 47 patients with prostate cancer with Gleason scores of 7–10 had low DHT levels in the prostate, as shown by univariate analysis [9]. In the current study, we found that low tissue DHT levels were associated with a high % positive cores.

Previous studies of tissue androgen measurements have had several problems, including small sample sizes, tissue-handling issues [15], problems with methodological accuracy and concerns about confounding factors. The tissue content of DHT decreases rapidly within 2 h at 37°C, and the tissue samples used in the present study were therefore frozen immediately (at -70°C) until analysis. To ensure the quality and precision of our measurement methods, we used prostate tissues spiked with various amounts of T or DHT to determine the precision of the LC/MS-MS method, and performed multivariate analysis after controlling for confounding factors.

Our results confirmed that high T levels in prostate tissue are related to high Gleason score, advanced clinical stage and a high % positive cores. Low DHT levels in prostate tissue were also associated with a high % positive cores. Thomas et al. reported that prostate-tissue expression levels of 5alpha-reductase type 1 and type 2 were increased in high-grade, compared with low-grade prostate cancer, which might explain the relationships between high T and low DHT prostate tissue levels and poor prognostic factors [16]. de Winter et al. reported that the intensity of androgen receptor immunostaining was decreased in more aggressive tumors [17], suggesting that androgen receptor heterogeneity might also offer an explanation of our findings.

Based on our results, we propose that prostate tissue T level measured by chemical biopsy may be a useful prognostic factor, in addition to Gleason score, clinical stage and % positive biopsy cores, for aiding the design of suitable therapeutic programs for prostate cancer. It is important that the tissue T level as a prognostic marker is determined simultaneously with the biopsy, to allow identification of the individual patient’s prognostic factors before making decisions about treatment options. Moreover, tissue T could help to subclassify Gleason 7 needle biopsies into a high-T and normal-T group, to distinguish between those patients with more aggressive disease and those with more indolent disease. This would be useful for selecting suitable patients for active surveillance.

Our results revealed that low serum T was associated with a Gleason score ≥8. In this regard, Garcia-Cruz et al. reported that low T was associated with high D’Amico classification, by multivariate analysis [18]. Hoffman et al. also reported that low free T indicated an increased risk of a biopsy Gleason score ≥8 (11% vs 0%, p = 0.025). Our results are compatible with the hypothesis that low serum androgen levels may influence poor-prognosis factors in prostate cancer [19], but no mechanism for this relationship has yet been established.

In our previous study, we first reported that CRPC may be predicted by prostate tissue T and DHT levels in a single biopsy specimen, obtained as a chemical biopsy, in patients undergoing cancer checkups, whether for prostate cancer or not [11]. Our present finding that high tissue T levels might be associated with poor prognostic factors may thus help predict prostate cancer aggressiveness.

The correlation of high tissue T levels with poor prognostic parameters (Gleason score, advanced clinical stage, high % positive cores) detected in this study suggests that tissue T may have a prognostic role, though no prognostic information was available in the current study. Further studies with larger numbers of patients and longer follow-up are warranted to confirm the validity of high tissue T levels as a prognostic marker.

## Conclusions

We conclude that high serum PSA levels and high tissue T levels in men with prostate cancer are significantly associated with indicators of poor prognosis, including advanced clinical stage, high Gleason score and % positive cores. Tissues T levels determined from biopsy specimens, in combination with pathological features, may be one of several useful diagnostic tools for predicting the prognosis and determining a suitable therapeutic program for patients with prostate cancer.

## Electronic supplementary material

Additional file 1: **Ethics committee.** (DOCX 11 KB)

## Abbreviations

LC-MS/MS: Liquid-chromatography tandem mass spectrometry
T: Testosterone
DHT: Dihydrotestosterone
PSA: Prostate-specific antigen
CRPC: Castration-resistant prostate cancer
RIA: Radioimmunoassay.
