# Supplementary material for: High testosterone levels in prostate tissue obtained by needle biopsy correlate with poor-prognosis factors in prostate cancer patients
Source: BMC Cancer. 2014 Sep 26;14:717. doi: 10.1186/1471-2407-14-717 (PMC4190297; doi:10.1186/1471-2407-14-717)
Supplement: Supplementary file 1 — Additional file 1: Ethics committee. (DOCX 11 KB) [file 12885_2014_4906_MOESM1_ESM.docx]

Additional file 1

This study was approved by the ethics committee of institutions described below.

Yokohama City University Graduate School of Medicine, Yokohama, Japan

Gunma University Graduate School of Medicine, Maebashi, Japan

Sapporo Medical University School of Medicine, Sapporo, Japan

Chiba University Graduate School of Medicine, Chiba, Japan

Kanazawa University Graduate School of Medical Science, Kanazawa, Japan

Mie University Graduate School of Medicine, Tsu, Japan

Osaka University Graduate School of Medicine, Osaka, Japan

Nagasaki University Graduate School of Biomedical Sciences, Nagasaki, Japan
